# Rhinitis Caseosa: A Forgotten Entity

**DOI:** 10.7759/cureus.71093

**Published:** 2024-10-08

**Authors:** Akshita Goyal, Tharun Rajeev, Manu Babu, Mayur Ingale, Rashmi P Rajashekhar, Shivangi Gupta

**Affiliations:** 1 Department of Otolaryngology, Head and Neck Surgery, Dr. D. Y. Patil Medical College, Hospital and Research Centre, Dr. D. Y. Patil Vidyapeeth (Deemed to Be University), Pune, IND

**Keywords:** antral cholesteatoma, fungal rhinosinusitis, nasal cholesteatoma, rhinitis caseosa, rhinolith

## Abstract

Rhinitis caseosa, also known as nasal cholesteatoma, is an uncommon disorder marked by the growth of caseous masses of material in the nose and, rarely, the maxillary antrum. This disease can be caused by tuberculosis, syphilis, erysipelas, Strepthrix alba infection, polyp degeneration, or suppuration due to protracted blockage. Patients mostly present with complaints of nasal obstruction with foul-smelling nasal discharge and can mimic features of sinonasal malignancy or fungal rhinosinusitis. Therefore, an early diagnosis and management become crucial. Herein, we present an unusual case of a female in her mid-20s with similar complaints. A diagnosis of rhinitis caseosa was made with a nasal endoscopy and computed tomography scan of the paranasal sinuses, which were eventually treated with surgical excision and confirmed by histopathological examination.

## Introduction

In 1874, Duplay coined the term "rhinitis caseosa," which was defined as a chronic inflammation of the nose associated with the formation of granulation tissue and the accumulation of fetid debris in the nasal cavity [1]. A few isolated cases have been reported in the past two decades, but the literature associated with this disease entity remains limited. Various theories in regard to its etiology have come up in the past, but the most widely accepted etiologies include tuberculosis, syphilis, erysipelas, aural cholesteatoma, polyp degeneration, or suppuration complicated by obstruction. A causative association between pulmonary tuberculosis and the pressure of the caseous material in the nose has been seen; the caseation is thought to be caused by degradation of the nasal epithelium caused by tubercle bacilli infection. Syphilis is associated with damage caused by the production and breakdown of gummata in the nose. The extension of erysipelas from the face to the nose produces epithelial desquamation, which results in the formation of a mass in one of the nasal passageways. Cholesteatoma that is similar to auditory cholesteatoma may be present. Caseous material can result from the degradation of mucosal polypi in the nose. The most widely recognized theory nowadays is that the phenomena of caseous rhinitis are due to the retention in the nose and sinuses of the products of suppuration owing to some sort of obstruction (e.g., rhinolith) under certain bacteriological and physical conditions that encourage its inspissation [2]. The term "rhinitis caseosa" seems to be disappearing from the otolaryngologic literature. There are several references to both Allergic Aspergillus sinusitis and allergic fungal sinusitis syndrome, where the stated clinical and radiological characteristics are extremely similar to rhinitis caseosa [3]. Hence the requirement for differentiating between the two is of utmost importance. The most commonly presenting symptoms are nasal obstruction, foul-smelling cheesy discharge from the nose, associated with blood, headache, and facial pain, depending on the anatomical site involved. They have bone-eroding properties but do not contain squamous epithelium, unlike cholesteatoma elsewhere. Typically, this disease can arise from anywhere in the body, the nose and paranasal sinus being the rarest. But when involved, the most frequently encountered sinus is the maxillary sinus. Due to the condition's rarity, there are no suitable management guidelines. Caldwell Luc's (Open) method has been proposed as the standard of care for maxillary sinus cholesteatoma [4]. In this case report, we discuss the presentation and management of a 24-year-old female with symptoms of rhinosinusitis, finally diagnosed as a case of rhinitis caseosa.

## Case presentation

A 24-year-old female patient presented with the chief complaints of intermittent left nasal discharge for two years. The nasal discharge was insidious in onset, gradually progressive, mucopurulent in nature, and foul-smelling. It was associated with recurrent upper respiratory tract infections. There was no history of epistaxis, facial pain, hyposmia/anosmia, or headache. No other significant positive history was present. Medical and personal history were non-contributory.

**Figure 1:**
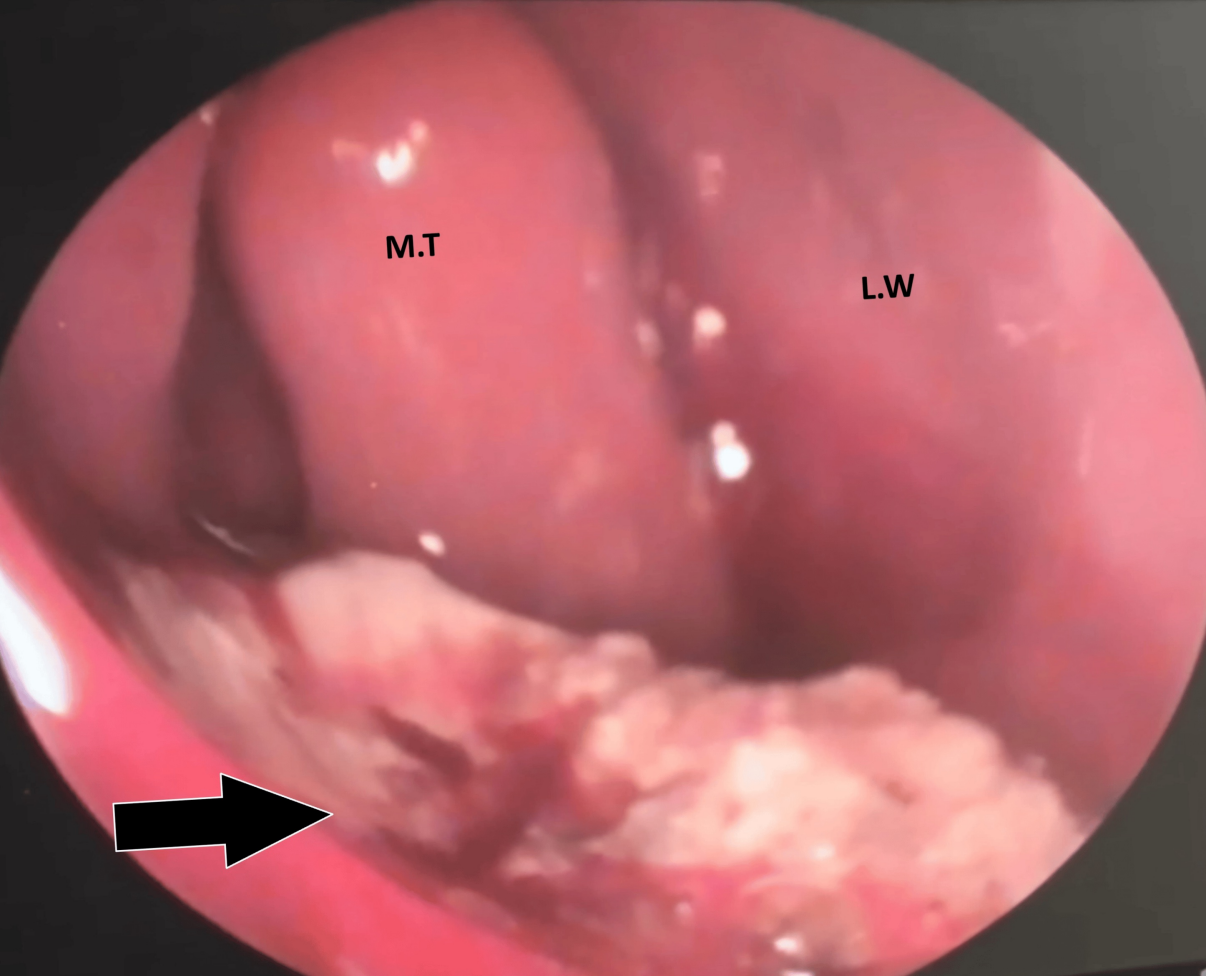
Diagnostic Nasal Endoscopy Image of the Left Nasal Cavity M.T: middle turbinate; L.W: lateral wall of the nose; the black arrow indicates cheesy white mass in the floor of the nasal cavity.

On clinical examination of the nose, there was an irregular white, cheesy mass on the floor of the left nasal cavity extending to the middle meatus. On probing, the mass was soft to firm in consistency, non-tender, and did not bleed on touch. There was no paranasal sinus tenderness, and the rest of the otolaryngologic examination was within normal limits. Diagnostic nasal endoscopy confirmed anterior rhinoscopy findings and showed that the inferior turbinate was atrophied and the nasal septum was pushed to the right side, revealing a roomy nasal cavity (Figure 1).

**Figure 2:**
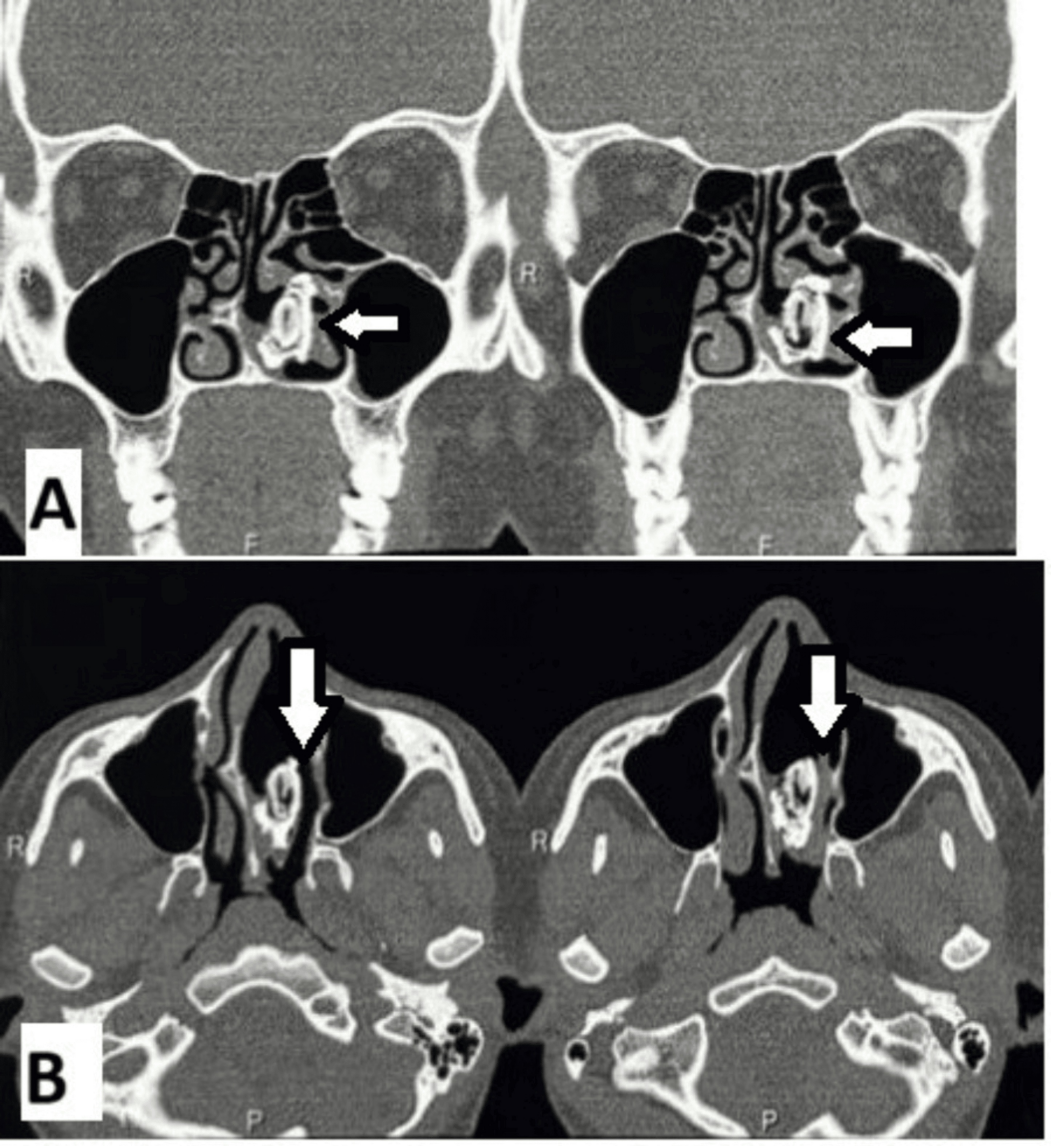
Computed Tomography Scans of the Paranasal Sinus (A) Coronal images of the paranasal sinuses show a radio-dense lesion (white arrow) abutting the inferior and middle turbinates. (B) Axial images of the paranasal sinuses reveal a radio-dense lesion (white arrow) measuring 24 x 12 x 17 mm within the left nasal cavity, laterally indenting the left inferior and middle turbinates and medially abutting the nasal septum. A septal deviation towards the right is also noted.

On the basis of history and examination, a provisional diagnosis of rhinitis caseosa with atrophic changes was made. Radiological evaluation was done with computed tomography scans of the nose and paranasal sinuses, which revealed a heterogenous oval radio-dense lesion of size 24 x 12 x 17 mm in the left nasal cavity, laterally indenting on the left inferior and middle turbinates and medially abutting the nasal septum. Widening of the left nasal cavity was noted and deviated nasal septum to the right, likely suggestive of a rhinolith (Figure 2).

**Figure 3:**
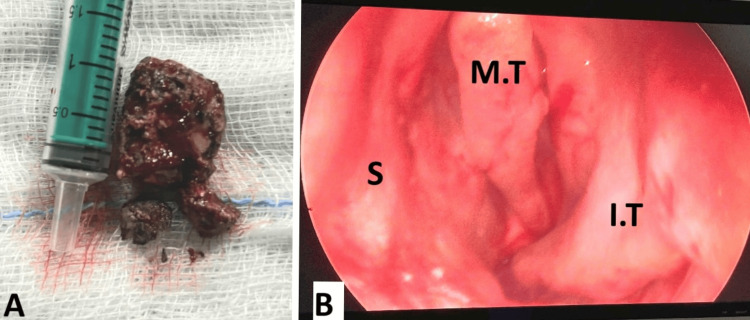
Postoperative Images (A) Mass excised as a whole. (B) Postoperative nasal endoscopic image of the left nasal cavity. M.T: middle turbinate; I.T: atrophied inferior turbinate; S: septum

On the basis of the above clinical and radiological findings, the patient was diagnosed with a case of rhinitis caseosa secondary to rhinolith obstruction and was posted for excision under general anesthesia with relevant consent. Intraoperatively, the mass was hard in consistency, attached to the middle turbinate, extending till the posterior choana. The specimen was removed in one piece and sent for histopathological evaluation. No anterior nasal packing was required and saline nasal drops and douching were started on postoperative day 1 (Figure 3).

**Figure 4:**
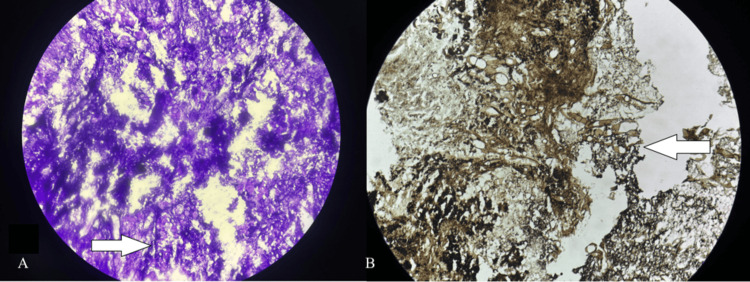
Histopathological Slides of Specimen (A) Hematoxylin and eosin stain showing acellular, amorphous tissue along with granular, calcified material, which was suggestive of rhinitis caseosa with fungal elements. The white arrow indicates fungal elements. (B) Grocott-Gomori’s Methenamine Silver (GMS) stain highlighting fungal elements. The white arrow points to the fungal structures.

Histopathological examination of the specimen revealed rhinitis caseosa with fungal septate hyphae with rhinolith (Figure 4).

Postoperatively, the patient was discharged on antibiotics, nasal drops, and saline nasal douching. Follow-up with the patient was uneventful.

## Discussion

Rhinitis caseosa, also known as nasal cholesteatoma, is characterized by the clinical condition of nasal polyposis and expansile rhinosinusitis. It has been regarded as the final stage of sinus inflammation. However, it is unclear if it is just a sign of rampant polyposis producing blockage to sinus drainage with stagnant secretions or if other mechanisms are involved [3]. Patients are usually of middle age, though extremes of age have been documented. In several instances, the obstruction has been attributed to a foreign body, a rhinolith, large septal crests or deviations, granulation tissue, tumors in the anterior part of the nose, gummas, septal hematomas, and perichondritis, and hypertrophic changes in the mucosa, particularly in the turbinates or chronic sinusitis. Mckenzie determined that the majority of patients were middle-aged, and that expansion of the nasal cavity was caused by pressure, similar to cholesteatoma in the ear. The most effective approach was to excavate the entire collection weekly. The nasal passageways were then sprayed with a mild solution of alcohol. He also concluded that he found malignant tumors lying behind caseous material [5]. Nasal cholesteatomas can be described as slow-growing benign tumors; they gradually increase in size, causing erosion of the surrounding bone without invading surrounding soft tissue structures [6]. Symptoms highly depend on the anatomical site involved. Extension of the lesion towards the osteomeatal complex causes rhinorrhea and sinusitis; as it erodes the anterolateral wall of the antrum, facial swelling becomes noticeable and tenderness increases [7].

A CT scan of the nose and paranasal sinus showing an expansile mass filled with air pockets should suggest the diagnosis of rhinitis caseosa [4]. A CT scan is preferred over magnetic resonance imaging, as the extent of soft tissue invasion in the bone is better visualized on a CT scan. Pathologically, the caseous mass is yellowish or gray-colored, foul-smelling, semi-solid in consistency, and resembles the contents of a sebaceous cyst. Histopathological examination often reveals pus cells and amorphous debris with traces of cholestrin, and fatty cells occasionally [2]. Cases where degenerate cells such as polymorphonuclear leucocytes, gram-negative bacilli, gram-positive cocci, and gram-positive filaments were present have been observed [8]. Treatment for this condition has been mainly attributed to surgical clearance, but clearance of the mass by means of Higginson's syringe with an Eustachian catheter has also been documented [9]. Complete surgical resection prevents the risk of recurrence, all the more so as cases of carcinous degeneration have been described [10]. In this case, the patient presented rather early with symptoms mimicking rhinosinusitis secondary to rhinolith, and we chose to surgically debride the mass endoscopically with no postoperative complications. Nasal passages were cleared with betadine solution, and the patient was started on nasal saline drops and douching on postoperative day 1 to prevent complications arising from a roomy nasal cavity like atrophic rhinitis. Antifungals were not indicated in this patient as the fungal elements were well localized within the lesion and no secondary changes were present in the nasal cavity and paranasal sinuses. Patients can be treated with oral or intralesional antifungals if features of fungal sinusitis persist even after mass excision. Patients can be followed up with a checkup every two weeks for the first three months, followed by once every six months for two years.

## Conclusions

The focus is now aimed at rhinitis caseosa, since its aggressive nature and proclivity for paranasal sinus and bone involvement make it an important component of our differential diagnosis for nasal masses. It is important that we distinguish it from cases of allergic fungal rhinosinusitis and sino-nasal malignancies. Utmost importance should be given to a complete surgical excision of the mass to avoid recurrence. In conclusion, rhinitis caseosa is a rare condition that develops as a complication of chronic nasal sepsis, often due to factors like obstruction and the thickening of secretions. While it can lead to severe symptoms such as nasal blockage, foul-smelling discharge, and even facial disfigurement if left untreated, the rarity of the condition is attributed to the specific combination of contributing factors. Early detection and treatment are crucial to prevent the progression of more severe complications.
